# Diving into a pool or volcano? Examining the influence of sentence context and task demands on sentence reading in younger and older adults

**DOI:** 10.1371/journal.pone.0279555

**Published:** 2022-12-30

**Authors:** Pennie Haigh, Naveen Hanif, Angela de Bruin

**Affiliations:** Department of Psychology, University of York, York, United Kingdom; Universidad Autónoma de Madrid, SPAIN

## Abstract

Cognitive ageing is often associated with slower lexical processing, which might influence both language production and comprehension. Words are typically used in context, which can further influence word processing and potential age differences. However, it remains unclear how older adults are affected by context during reading. Older adults are reported to have in-tact semantic knowledge, which could potentially help them to process words predicted by semantic information in the preceding context. However, potential difficulties with semantic control might mean older adults have greater difficulty suppressing interfering information from mismatching contexts. In this study we examined the influence of contexts that either predicted a specific target word (“matched”, e.g., “*The man watched the lava erupt from* ***the volcano***”) or predicted another word than the target (“mismatched”, e.g., “*The swimmer dived into* ***the volcano***”) as compared to neutral contexts (e.g., *“They went to see* ***the volcano”****)*. We also examined the potential role of task demands by asking participants to either just read the sentences for comprehension or to answer questions. Forty younger adults (18–35 years old) and forty older adults (65–80 years old) completed a self-paced reading task in which we measured reading times for the target words. Older adults showed slower reading times overall. Matched sentence contexts facilitated reading times in both age groups. Surprisingly, mismatched sentence contexts did not hinder reading times in either age group. Furthermore, reading times were not influenced by task demands. Together, this shows the importance of studying language in context. While interference from mismatching sentence contexts might have not been substantial enough to delay reading, reading was faster when processing expected words. This suggests older adults can indeed benefit from semantic knowledge to facilitate word processing during comprehension. This occurred even when no additional task was presented and people were purely reading for comprehension.

## 1. Introduction

Word-finding difficulties are among the most annoying hindrances associated with cognitive ageing [1], which impacts engagement in social interactions [2]. With social interaction being important for physical and mental wellbeing, it is vital to understand language changes, and potential difficulties, in later life. Previous research has shown that age influences language production (e.g., [3]) and comprehension (e.g., [4]). For example, older adults experience more tip-of-the-tongue moments (failing to retrieve a word from memory despite knowing some of the word’s characteristics) and slower word retrieval (knowing a word but needing more time to access it). However, words are rarely used in isolation, making it important to study language in context. Previous research assessing ageing and context effects on language comprehension has shown mixed outcomes [4]. Furthermore, the type of task used, and the task demands that come with it, might influence age effects. This study therefore examined if and how sentence contexts and tasks modulate age effects during language comprehension.

### 1.1. Effects of sentence context on word processing

In younger, and to some extent older adults, there is a vast amount of studies assessing the effect of sentence context on word processing. Research has found that constraining sentence contexts (which predict a specific word, for example “The farmer milked **the cow**”) can facilitate word processing compared to non-constraining sentence contexts (contexts that do not prime a specific word, for example “The man saw **the cow**”). For example, one study used a picture-naming task in which participants were asked a question and answered with the name of a picture, whilst ignoring an additional distractor word [5]. The question formed either a constraining or non-constraining context towards the picture answer. Constraining contexts facilitated picture naming more than non-constraining contexts as participants were able to use the semantic information from the sentence context to prepare target word retrieval and to reduce semantic interference from the distractor. In terms of language comprehension, this area has mainly been investigated through the use of EEG (electroencephalography), focusing on the N400 (an ERP component related to processing the meaning of words). Several EEG studies have shown a reduced N400 component in response to target words in constraining sentences as compared to non-constraining sentences (e.g., [6–8]). This suggests that constraining sentences help listeners or readers to prepare for an upcoming target word that is in line with the context. Research has furthermore shown that context can influence reading times too, by using a self-paced reading task in which participants read a longer story at their own pace [9]. Reading became faster as participants started building up a global context based on the preceding discourse, suggesting that participants were facilitated by the semantic information presented in context during language comprehension. Further research suggested that this might also be the case when processing non-literal language [10]. Participants were presented with metaphor-relevant (Prime: “The lawyer for the defence is a shark” > Target: “*Sharks are tenacious*”) or irrelevant (Prime: “The lawyer for the defence is a shark” > Target: “*Sharks are good swimmers*”) contexts and had to indicate whether the sentences made sense. Response times, relative to literal sentences, were faster for metaphor-relevant contexts but slower for metaphor-irrelevant contexts. Together, these findings provide strong evidence that constraining, predictable contexts can facilitate both language production and comprehension. Such facilitation might arise as a consequence of semantic priming of words related in meaning (e.g., “milked” enhancing activation of “cow”) and/or due to listeners using contextual information to predict upcoming words (e.g., [6]).

Conversely, words that are incongruent with the preceding sentence context (“mismatch”) can also modulate language processing. Studies using picture-word interference paradigms, for example, typically ask participants to generate a word describing a picture while ignoring another written word. Word retrieval is usually slower when the distractor is related in meaning (e.g., “cat” when saying “dog”) than when the distractor is unrelated (e.g., “bed”; see e.g., [11]). When reading sentence contexts, this interference can be present too. For example, one study presented participants with sentences that included either an expected word (“Dutch trains are *yellow*”), an unexpected word that violated world knowledge (“Dutch trains are *white*”, which is unexpected given that Dutch trains are typically yellow), or an unexpected word that formed a semantic violation (“Dutch trains are *sour*”) [12]. Both words violating world knowledge and those forming a semantic violation elicited larger N400 effects than expected words. This suggests that words mismatching sentence context (either through predictions based on semantics or based on world knowledge) can hinder language processing, possibly because more likely, competing words are activated more strongly than the unexpected target word.

### 1.2. Semantic knowledge and control in older adults

Sentence context can thus influence language processing. However, it is an open question how context influences language processing in older adults. Research has highlighted that older adults have intact semantic knowledge, which could suggest that they might still be able to benefit from this knowledge when processing language in constraining contexts that match the target word. For example, researchers asked older and younger adults to complete a lexical decision task (classifying stimuli as words or non-words) and a synonym matching task (selecting the synonym of a given word). Older adults performed better than younger adults on both tasks, suggesting that they have a greater depth of semantic knowledge [13]. Further research found similar evidence when conducting three tests of word production and two tests of word knowledge with older and younger adults [14]. Although younger adults outperformed older adults during tests of production (suggesting older adults had greater difficulty retrieving words), older adults performed better than younger adults during tests of vocabulary, suggesting they knew more words. Supporting research used a multiple choice vocabulary test, in which older and younger adults were shown a target noun and were asked to select the correct interpretation of the noun from the given answer options [15]. These results too found that older adults performed better than younger adults. These various studies thus confirm that older adults have intact (and potentially even stronger) semantic knowledge, which they might be able to benefit from when processing language in context.

However, research has also highlighted that although older adults have intact semantic knowledge, they may have reduced inhibitory and/or semantic control, which might make it more difficult for older adults to suppress any interference from incongruent semantic contexts when processing language. For example, older adults have been found to experience difficulty suppressing task-irrelevant associations between words [13]. Participants were asked to connect words based on, for example, their colour (e.g., “salt” and “snow”). During congruent trials, the correct response was also related in meaning (snow-cloud). During incongruent trials, the correct response was not related in meaning (salt-snow) while one of the distractor words was (e.g., “pepper” had to be ignored). Older adults performed more poorly in the incongruent condition than younger adults. This suggests that in the incongruent condition, older adults had greater difficulty identifying and responding to relevant associations in the presence of semantically competing information [16]. Picture-word interference tasks too have shown a larger interfering effect of semantically related distractors in older than younger adults (e.g., [17]). This reduced ability to inhibit irrelevant information could affect performance on a wide range of cognitive processes and tasks (e.g., [18]), although this inhibitory deficit might show task-specific effects too (e.g., [19]).

Therefore, although older adults might benefit from semantic knowledge when it is compatible with their goal (e.g., processing a target word), they may not be able to prevent interference stemming from irrelevant information as well as younger adults during lexical processing that is incompatible with a sentence context.

### 1.3. Sentence context and word processing in ageing

With regards to empirical evidence assessing context and word processing during language comprehension throughout ageing, research focusing on the sentence level has provided mixed outcomes (see [4] for a full literature review). Some studies have suggested that older adults experience greater facilitation from constraining contexts that are semantically congruent than younger adults. Several studies asked older and younger adults to complete a self-paced reading task, in which participants read at their own pace while word-by-word reading times were measured ([9, 20]). Reading times decreased when contextual support was presented (as opposed to single sentences), with a stronger context effect found for older adults. This suggested that constraining contexts provided greater facilitation for older adults than younger adults during comprehension. Further evidence consistent with this finding presented older and younger participants with ambiguous texts either with or without a title [21]. The addition of the title provided further contextual information, which was found to provide greater facilitation for older adults than younger adults during comprehension.

Other research has suggested that older adults can still use context to facilitate comprehension, but that they benefit from this to the same extent as younger adults. One study presented older and younger participants with a series of either predictable or unpredictable sentences and tracked their eye movements during reading [22]. Fixation duration times decreased with increasing predictability amongst both age groups, suggesting that context facilitates reading in both age groups to the same extent. This is further supported by research in which older adults and college students read stories followed by true or false statements that either matched or mismatched the context of the story [23]. Older adults were able to use contextually relevant information as well as younger adults to facilitate language comprehension. Similarly, both younger and older adults showed increased facilitation when target words were placed within context [24] or when processing metaphor-relevant contexts relative to literal sentences [10]. This suggests that both age groups can be facilitated equally by relevant contexts during language comprehension.

However, other studies have suggested that older adults cannot benefit from semantic information as much as younger adults (smaller context facilitation effect for older adults). One EEG study recorded the N400 responses of younger and older adults who read sentences which either formed constraining or non-constraining contexts [6]. Older adults showed the expected N400 effect (reduced N400 for constraining sentences), but the difference between constraining and non-constraining contexts was smaller for older adults than younger adults. Later electrophysiological evidence supported this finding by also showing that older adults’ processing was influenced by a constraining sentence context, but to a lesser extent than younger adults [7] and by showing that older adults displayed delayed and reduced N400 effects compared to younger adults [8]. These studies suggest that older adults did not benefit from the constraining contexts by predicting upcoming words as much as younger adults.

When comparing constraining and non-constraining contexts, evidence regarding the effects of sentence context on language processing in older adults is thus mixed. While most studies seem to suggest that older adults can benefit from constraining contexts that are semantically congruent with and/or prime a target word, it is unclear whether older adults benefit less, to the same extent, or more than younger adults. In other words, while older adults seem to have intact semantic knowledge, it remains unclear whether this semantic knowledge can help them to overcome age-related slowing of lexical processing during comprehension.

Furthermore, most studies have compared constraining contexts or expected words to either no context at all or to different types of “non-constraining” contexts or “unexpected” words. The non-constraining contexts are often constructed to be neutral and not strongly predictive of any target word (e.g., [6]). In other studies, constraining contexts with expected target words are compared with unexpected target words (e.g., [8]). As described above, older adults might have difficulties suppressing interfering information. This might also influence language comprehension. For example, processing of implausible sentences (such as “because the light is on, the room is dark”) was found to be more difficult for older adults [25], although others suggested that this type of plausibility effect is comparable for younger and older adults [26]. It is therefore important to not just consider constraining sentence contexts that predict the target word (which might facilitate language processing) but to also consider sentence contexts that predict other words than the target (which might increase interference and thus hinder language processing). When expected and unexpected words are compared directly without a neutral, non-constraining baseline (e.g., [8]), however, it is difficult to tease apart the potentially facilitating role of “matching” contexts (that predict the target) and the potentially interfering role of “mismatching” contexts (that predict other words). We therefore included a neutral sentence condition in which the sentence context did not predict a specific word. We compared constraining “matching” (predicting the target word) and constraining “mismatching” (predicting another word) contexts to these neutral contexts to help to understand how older adults might use and manage both facilitating and interfering semantic contexts.

### 1.4. Current study

The current study thus aimed to better understand word processing during sentence reading in younger and older adults, which so far has produced mixed results in the literature. Furthermore, previous research has often focused on the effect of constraining, matched contexts, but has not always distinguished between neutral contexts and mismatching contexts that predict another word (cf. [4]). Our study separated those contexts, to directly compare the facilitating versus interfering effects sentence contexts might have on word processing. Rather than using mismatching sentence contexts that violate world knowledge (e.g., it being dark because the light is on [25]), we studied sentence contexts in which a target word was unlikely to occur but not impossible to happen in real life. Participants completed a self-paced reading task, which measured their reading times of a target word within one of three sentence contexts: matched (e.g., “The farmer milked **the cow** early in the morning”); mismatched (e.g., “The parents went to the pet shop to buy **the cow** for their son”); or neutral (e.g., “They took a picture of **the cow** on their day out”). This assessed whether sentence contexts modulate reading times as well as potential differences between age groups.

Mixed outcomes within the literature could furthermore also be due to the different ways in which participants have been tested, with some studies simply assessing reading comprehension (e.g., [6–8]) and others requiring participants to make lexical decisions (e.g., [13]). These tasks may place different levels of demand onto an individual’s cognitive load and depth of language processing, which might be lower when participants read for comprehension than when they are asked to respond to the information presented in the sentences. This could potentially influence age effects, which might be more pronounced when cognitive load is high (e.g., [25]). The current study therefore also aimed to determine whether task demand influences age effects, by asking participants to either read simply for comprehension or to answer questions after reading each sentence.

Based on previous literature (e.g., [27]), it was hypothesised that there would be a main effect of age, with older adults having slower reading times overall than younger adults when reading the target word. In line with previous research (e.g., [5]), it was also predicted that reading times of target words presented in matched contexts would be shorter than in neutral contexts (“match effect”). Given that the mismatched context presented semantic information predicting another target word, we expected reading times of target words to be longer in mismatched than in neutral contexts (“mismatch effect”).

For the interaction between age and sentence context, we considered that there were three possible outcomes. Starting with the match effect, one possibility was that older and younger adults would benefit equally from words being predictable in the sentence context (similar match effect) [22]. This would suggest that both older and younger adults are able to use semantic knowledge to benefit from matching contexts to improve language processing to the same extent. Alternatively, a larger match effect for older adults (e.g., [20, 21]) would suggest that older adults are able to use semantic knowledge to benefit from matching contexts to improve language processing more than younger adults and to potentially overcome slower lexical processing. The third possibility is a smaller match effect for older adults (e.g., [6–8]), which would suggest that older adults are not able to use their semantic knowledge, or use it less effectively, to benefit from matching contexts to improve language processing as well as younger adults.

Regarding the mismatch effect, the first possibility is that mismatched sentence contexts hinder comprehension (indicated by slower reading time) for both age groups more than neutral contexts (“mismatch effect”). This would suggest that both older and younger adults are influenced negatively by the need to inhibit irrelevant semantic information in mismatching contexts. Alternatively, there may be a larger mismatch effect for older adults than younger adults, as supported by previous research (e.g., [16, 17]). This would suggest that older adults are not as capable as younger adults at inhibiting semantic knowledge to prevent mismatching contexts hindering their processing speed. The third possibility is that there may be a smaller mismatch effect for older adults than younger adults. This would suggest that older adults may not use their semantic knowledge as much as younger adults during comprehension (and possibly do not form predictions), so mismatching contexts might not hinder their processing speed as much.

Task demand was an exploratory variable within the study. We predicted that older adults would show slower reading times than younger adults when completing a task (answering questions) in addition to the self-paced reading than when they were just reading the sentences (without an additional task). The study also examined whether task demands modulate potential age effects within the match and mismatch effects described above.

## 2. Materials and methods

This study was pre-registered on the Open Science Framework: https://osf.io/na6vy/.

### 2.1. Participants

A volunteer sample of 80 participants was recruited through Prolific.co and received monetary compensation for their participation. Given that it was difficult to determine an exact effect size for this type of task based on previous studies, we conducted a power analysis with an estimated medium-sized effect of f = 0.25 for an interaction between age and sentence context. Using G*Power, this analysis showed that 80 participants were sufficient to achieve over 80% power to detect an interaction of this size. Ethical approval was obtained from the Department of Psychology at the University of York. Participants were aged between 18–35 or 65–80 years old and were native monolingual English speakers. They did not use a hearing aid and had normal or corrected-to-normal vision. Participants had not used medication that affected their concentration in the last three months and did not have a neurodegenerative/cognitive impairment or a language/reading disability. Given that the study was conducted online, we were not able to use an assessment of cognitive functioning such as the MMSE. However, we used Prolific’s screening criteria to only invite participants without a history of head injury, cognitive impairment, or dementia, which participants also confirmed in our questionnaire. An additional three participants completed the study but were excluded as two did not meet the criteria described above and one did not complete the study correctly. Additionally, we assessed whether participants met our attention checks. No participants had reading times of less than 200ms and all participants scored well above 70% accuracy on the questions asked after reading the sentences.

The two age groups were matched on gender ratio, education level (highest level of education achieved, although the older adults completed, on average, approximately two years of education less than the younger adults), and handedness. Within the group of 40 older adults, the mean age was 68.78 years old (*SD* = 3.58), with 23 female and 17 male participants. In terms of education, 22 participants had a graduate-level education and the average number of years in education was 13.77 (*SD* = 3.30). Amongst the 40 older adults, 35 were right-handed, four were left-handed, and one was ambidextrous. Within the group of 40 younger adults, the mean age was 23.95 years old (*SD* = 5.22), with 23 female and 17 male participants. In terms of education, 22 had a graduate-level education and the average number of years in education was 15.69 (*SD* = 2.12). Amongst the 40 younger adults, 36 were right-handed and four were left-handed.

### 2.2. Design

The study’s main task was a self-paced reading task using a mixed design, containing both within- and between-subject variables. The first independent variable was sentence context, which contained three levels: matched (sentence predicting the target word), mismatched (sentence predicting another word than the target), and neutral (sentence not predicting one specific target word). All participants were exposed to all three types of sentence context (within-subject). The second independent variable was task demand, which contained two levels: reading sentences for comprehension only and reading sentences before answering questions. Participants were exposed to both types of task demand (within-subject). Age group was a between-subject variable, with participants categorised as either younger adults (18-35yrs) or older adults (65-80yrs).

The dependent variable was reading time (ms) as a measure of processing speed (i.e., the time interval between presenting the target word and participants pressing the spacebar).

### 2.3. Materials

The main task participants completed was a self-paced reading task in which they saw target words in sentence contexts. Sentence stimuli belonged to one of three context conditions: matched, mismatched, or neutral. Matched sentence contexts primed the target word (e.g., “The farmer milked **the cow** early in the morning”). Mismatched sentence contexts contained words before that target word that did not prime the target word and instead primed a different word (e.g. “The parents went to the pet shop to buy **the cow** for their son”). We created mismatched sentences that were unlikely to happen but not semantic anomalies that are impossible to occur (i.e., while it is unlikely to find a cow in a pet shop, it is not an impossible event). Neutral sentence contexts contained words before the target word that could be followed by the target word but also by many other words (e.g. “They took a picture of **the cow** on their day out”). Words after the target word did not contribute to the context of the sentence (i.e., they could not influence target-word processing), but we always made sure the target word was not the last word in the sentence to prevent the reader from slowing if they had seen a full stop after the target word.

To assess whether the three different types of contexts indeed differed in the likeliness of the target to occur in that sentence context, participants also rated how likely they thought the target word was to be found in each sentence (on a scale from 1 to 7: 1 = very unlikely; 7 = very likely; only presenting the sentence parts preceding the target word). The likeliness ratings confirmed that the sentences formed the correct context for each condition, with target words being most likely to be found in matched contexts and least likely in mismatched contexts (see “Results” for an analysis of these ratings). Furthermore, many of the stimuli were based on stimuli created for another study ([28]) in which similar likeliness ratings were provided by a separate group of participants who confirmed targets were most likely in matched contexts and least likely in mismatched contexts.

A total of 60 sentence groups were created (see S1 File), with each group including three sentences corresponding to the three contexts. Each participant thus responded to 180 sentences, with a target word presented three times, once in each context. Each sentence group was combined with two possible target words (see Table 1), with a given sentence forming a match with one target word but a mismatch with the other target word (e.g., “The farmer milked **the cow…**” versus “The farmer milked **the hamster…**” and “The parents went to the pet shop to buy **the cow…”** versus “The parents went to the pet shop to buy **the hamster…**”). Half of the participants saw one set of the target words with the sentences in one type of context (e.g.,”The farmer milked **the cow…**” as the matched sentence and “The parents went to the pet shop to buy **the cow…**” as the mismatched sentence) and the other half of the participants saw the same sentences but with the other target words in the opposite context (e.g., “The farmer milked **the hamster…**” as the mismatched sentence and “The parents went to the pet shop to buy **the hamster…**” as the matched sentence). The neutral sentence maintained the same context between sets (i.e., half of the participants saw “They took a picture of **the cow…”** and half saw “They took a picture of **the hamster…**”.). This ensured that across participants, the same sentences occurred as both matched and mismatched contexts, thus excluding potential differences between match and mismatch effects as a consequence of the stimulus materials used. By presenting each sentence once per participant, we ensured that the participant only saw the same sentence once, thus avoiding any effects of sentence predictions. Each target word, however, was presented once per context to each participant to exclude any potential effects of contexts resulting from target words differing in reading times.

**Table 1:** Example of a sentence pair. Half of the participants would see the three sentences with “cow” and the other half would see the three sentences with “hamster”. A given sentence formed a matched context for one target and a mismatched context for the other target. Sentences never ended with the target word, but for simplicity, we removed the sentence endings from these examples.

	Matched	Mismatched	Neutral
**Target**			
*Cow*	The farmer milked **the cow**…	The parents went to the pet shop to buy **the cow…**	They took a picture of **the cow…**
*Hamster*	The parents went to the pet shop to buy **the hamster…**	The farmer milked **the hamster…**	They took a picture of **the hamster…**

Sentence length was matched across contexts so that sentences had an average total of 10 words (Match/Mismatch *M* = 9.9, *SD* = 1.67; Neutral *M* = 9.6, *SD* = 1.82), with an average of 5 words before the target word. Sentences were displayed in parts (i.e., multiple words at a time, for example “The farmer milked”), with the target word always presented on its own. Each target word was preceded by either one or two sentence parts. Log frequency of the other words in the sentence preceding the target was calculated using N-Watch [29]. The subjects of the sentences had an average log frequency of 1.62 (*SD* = 1.01) for matched/mismatched contexts and 1.90 (*SD* = 1.03) for neutral contexts. The main verbs had a mean log frequency of 1.77 (*SD* = 0.75) for matched/mismatched contexts and 1.94 (*SD* = 0.68) for neutral contexts. There were no significant differences between the contexts in terms of overall sentence length, number of words or parts before the target word, or average frequency of the subjects and verbs of the sentences. Where possible, the definite article ‘the’ was used before the target word in all sentences.

To assess the effects of task demand, one condition included questions based upon the sentences that participants read. A question was created for each sentence that asked about either the target word (e.g., What did the farmer milk?) or the context (e.g., Who milked the cow?) of the sentence. The conditions were matched on the number of questions of each type (asking about target word or context; see S1 File). We created different types of questions so that participants did not know what to expect (and thus had to pay attention to both the sentences and the targets), but did not aim to compare these different question types. Each question had three answer options. For sentences asking about the context these three options were all plausible answers. For sentences asking about the target, two answer options were the target words (matched and mismatched) and one option was plausible and semantically related to the question.

### 2.4. Procedure

The experiment was conducted on Gorilla.sc [30]. Participants read an information sheet and completed a consent form to confirm that they met the criteria and agreed to participate. A background questionnaire determined whether participants met the criteria and provided further demographic information (see “Participants”).

Participants were allocated to one of eight experiment lists. Half of the participants in each age group first completed the task with questions and the other half completed the task without questions first. Half of the participants saw the sentences with the first target word, and the other half of participants saw the sentence with the second target word of the pair. Within each age group, 21 participants saw one half of the sentences with a question, and 19 participants saw the other half of the sentences with a question. Participants were instructed to read the parts of the sentences as quickly as possible while still making sure they understood the content before pressing the spacebar to see the next part of the sentence. For the task with questions, participants were shown a question after every sentence and selected an answer from a choice of three options. For the task without questions, participants saw a screen telling them to press the spacebar to continue after each sentence. At the start of each task, participants completed three practice trials and they were given a break half way through the main trials.

After each reading task (with and without questions), participants also completed the NASA-TLX [Task Load Index, 31], and after finishing both parts they completed an overall NASA-TLX for the entire reading experiment. The NASA-TLX is widely used to measure the subjective experience of workload. Participants were asked to indicate how they experienced the task in terms of mental demand (how mentally demanding was the task?), physical demand (how physically demanding was the task?), temporal demand (how hurried or rushed was the task?), performance (how successful were they in accomplishing the task?), effort (how hard did they have to work to achieve that performance?), and frustration (how insecure, discouraged, irritated, stressed, and annoyed were they?) on a scale of 1–100 (1 = very low; 100 = very high). At the very end, when completing the overall NASA-TLX, they were also asked to indicate which of those experiences they found more important when describing the experienced workload. This was assessed by providing participants with every possible combination of experiences (e.g., mental and physical demand) and asking them to select the experience they found most important for each comparison.

Following the reading task and NASA-TLX, participants also completed a likeliness-rating task to rate how likely each target word was to occur in the sentence contexts (see “Materials”). Participants only evaluated the sentences that they had seen within the self-paced reading task. The order of this procedure prevented the likeliness-rating task from influencing participants’ reading times during the self-paced reading task. The experiment lasted approximately 30 to 45 minutes in total.

### 2.5. Data analysis

The data are available at: https://osf.io/na6vy/. Analyses were conducted with SPSS Version 27.

Likeliness ratings were examined using a 2x3 ANOVA (Age: younger, older; Context: matched, mismatched, neutral), with pairwise comparisons (Bonferroni corrected) used to confirm that each sentence context differed from one another in the likeliness of the target word in a given sentence. Sphericity was violated and Greenhouse-Geisser corrections are therefore reported.

For the self-paced reading task, RT outliers more than 2.5 *SD* above or below the mean per participant, sentence context, and task demand were removed, as well as responses that were faster than 200ms. This was conducted using the trimr package [32]. The results were then analysed using a 2x3 ANOVA to determine whether there was a context effect, age effect, or interaction between the two variables. If a context effect was found, a pairwise comparison was used to establish where the effect resided within the three levels of the independent variable. A Bonferroni correction was used during the pairwise comparison to account for multiple comparisons. Given that data were not normally distributed, we also analysed log-transformed RTs (which were normally distributed in almost all conditions). The results from these ANOVAs were the same as the analysis with the untransformed RTs, and for simplicity, we just report the analysis with the untransformed RTs below. The first analysis collapsed across task demands (with/without questions) while a second analysis was conducted to also include task demand as an additional variable. Given that the mean accuracy scores of the answers to questions were above 95% correct, we did not analyse these further.

Workload effects from the NASA- TLX were calculated by counting how often participants chose each experience as most important between two comparison options (e.g., how often they said they found "frustration" the most important compared to another experience). The raw score for each experience was computed and multiplied by the number of times it was chosen as most important. All weighted experiences were summed up and divided by the total number of comparisons participants had to choose from. Two analyses were run, one assessing overall workload experience (indicated after the reading task) and one assessing workload after each part of the reading task (with and without questions). An independent t-test was conducted to determine whether there was a difference in overall workload experience between older and younger adults. A mixed ANOVA compared the workload assessments after each part of the reading task to establish whether there was a significant difference in workload between the tasks with and without questions, and whether there was a significant interaction between age and task demand.

## 3. Results

### 3.1. Likeliness ratings

The likeliness ratings (Table 2) showed that target words found in matched sentence contexts were rated to be most likely, followed by neutral sentence contexts, with target words in mismatched sentence contexts being rated as the most unlikely. This result was confirmed in a mixed 2x3 ANOVA, which found a main effect of context (*F*(1.664,129.789) = 912.000, *p* < .001, η_p_^2^ = .921). All pairwise comparisons were significant (*p* < .001), which showed that the matched context differed significantly from the neutral context, the mismatched from the neutral context, and the matched from the mismatched context. There was no main effect of age (*F*(1,78) = 2.084, *p* = .153, η_p_^2^ = .026) but there was an interaction (*F*(1.664,129.789) = 4.133, *p* = .024 η_p_^2^ = .050). This was due to younger and older adults’ scores differing slightly in the neutral and mismatched ratings, however independent t-tests showed that this was a slight but not significant difference (matched: *p* = .388; mismatched: *p* = .056; neutral: *p* = .088). Additional ANOVAs per age group confirmed that the sentence contexts differed significantly in each age group individually, with a main effect of context in both younger adults (*F*(1.569,61.190) = 290.363, *p* < .001, η_p_^2^ = .882) and older adults (*F*(1.408,54.909) = 833.819, *p* < .001, η_p_^2^ = .955). All pairwise comparisons between contexts remained significant (*p* < .001). These analyses thus confirm that in both age groups target words were most likely to be found in matched sentence contexts and least likely in the mismatched sentence contexts.

**Table 2:** Mean likeliness score (scale 1–7) and standard deviation for each context in each age group.

	Younger adults	Older adults
**Likeliness rating**		
*Matched*	6.40 (0.69)	6.50 (0.34)
*Neutral*	3.95 (1.04)	3.57 (0.91)
*Mismatched*	2.79 (0.91)	2.47 (0.52)

### 3.2. Self-paced reading task

Accuracy in response to the comprehension questions asking about the sentences was high in all sentence contexts for both older adults (Matched *M* = 99.3; *SD* = 1.7; Neutral *M* = 99.4, *SD* = 1.8; Mismatched *M* = 98.5, *SD* = 2.5) and younger adults (Matched *M* = 99.3; *SD* = 1.4; Neutral *M* = 97.7, *SD* = 3.4; Mismatched *M* = 97.8, *SD* = 3.0). All participants scored over 93% correct, confirming they paid attention to the sentences they were reading.

**Fig 1:**
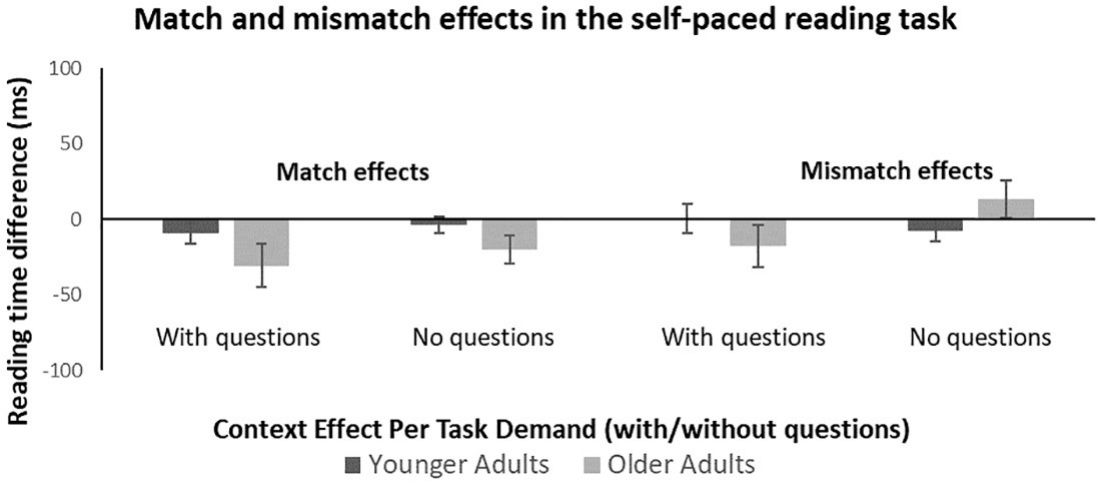
Match and mismatch effects in the self-paced reading task. Match (left) and mismatch effects (right) from the self-paced reading task. Match effects are computed as the difference in reading times between matched and neutral contexts, with negative scores showing a larger facilitation (faster reading during matched contexts). Mismatch effects are computed as the difference in reading times between neutral and mismatched contexts, with positive scores showing more hindrance (slower reading during mismatch contexts). Effects are shown for the part of the task with questions (left) and for the task just requiring reading for comprehension (no questions, right). Darker bars show results from younger adults and lighter bars from older adults. Error bars reflect +/- 1 standard error of the mean.

As pre-registered, we analysed the reading times in the self-paced reading task in two steps. The first analysis just included age and sentence context as variables (collapsed across the parts with and without questions). The self-paced reading task showed that reading times for matched sentence contexts were fastest, followed by mismatched sentence contexts and neutral sentence contexts (see Table 3). This result was confirmed in the mixed 2x3 ANOVA, which found a main effect of context (*F*(2,156) = 4.830, *p* = .009, η_p_^2^ = .058). A pairwise comparison (Bonferroni corrected) showed that reading times were significantly different between matched and mismatched contexts (*p* = .045) and between matched and neutral contexts (*p* = .008, see Fig 1). However, reading times were not significantly different between mismatched and neutral contexts (*p* > 0.999, see Fig 1). There was a main effect of age (*F*(1,78) = 34.983, *p* < .001, η_p_^2^ = .310), reflecting that younger and older adults differed significantly in their reading times across sentence contexts, with older adults being slower than younger adults (see Table 3). There was no interaction (*F*(2,156) = 2.037, *p* = .134, η_p_^2^ = .025) as the effect of sentence context (i.e. the facilitating effect of matched sentence contexts but no effect of mismatched sentence contexts, see Fig 1) did not differ significantly between the two age groups. Given the large difference in overall RTs between younger and older adults, we also z-scored the data. Analyses on the z-scored data also showed a main effect of context but no interaction between context and age.

**Table 3:** Mean reading time (ms) and standard deviation per sentence context in the parts with and without additional questions in each age group.

			Younger adults	Older adults
**Task Demand: With Questions**				
*Questions*		*Matched*	520.50 (150.70)	822.03 (295.81)
*Questions*		*Neutral*	529.74 (147.31)	852.86 (316.23)
*Questions*		*Mismatched*	530.16 (148.77)	834.83 (293.09)
**Task Demand: Without Questions**				
*No Questions*	*Matched*		563.44 (167.25)	892.81 (454.42)
*No Questions*	*Neutral*		567.31 (175.97)	912.71 (452.07)
*No Questions*	*Mismatched*		559.87 (169.11)	925.84 (481.92)

To match the conditions in terms of target-word processing, we repeated each target three times. Given that reading times might be influenced by repetition, as an exploratory check, we reran the analysis including repetition (first, second, or third target-word presentation) as a variable. In this analysis, we excluded one participant whose mismatch responses on the third repetition fell six *SD* above the group mean. This outlier was likely a consequence of splitting the data into three repetition conditions resulting in a low number of trials, which were strongly influenced by a few particularly slow trials (given that there was no time limit). Reading times decreased with target-word repetition (*F*(1.294,99.650) = 56.201, *p* < .001, η_p_^2^ = .422). This interacted with age (*F*(1.294,99.650) = 8.148, *p* = .003, η_p_^2^ = .096), such that older adults’ reading times benefited more from word repetition. Finally, repetition also interacted with sentence context (*F*(3.375,259.849) = 3.087, *p* = 0.023, η_p_^2^ = .039). This was driven by the mismatch trials. While the match and neutral trials were read the fastest when the word was presented the third time, the mismatch trials were faster in the second (*M* = 683, *SD* = 260) than first presentation (*M* = 738, *SD* = 296) but did not decrease further when presented the third time (*M* = 677, *SD* = 258). Crucially for our main questions of interest, however, the effects of context (*p* < .05) and age (*p* < .001) remained significant while the interaction between age and context was not (*p* > .20).

#### 3.2.1. Task demand

When including task demands (sentences read with or without questions) in a 2x3x2 ANOVA, the main effect of context was still present (*F*(2,156) = 4.726, *p* = .010, η_p_^2^ = .057), as well as the main effect of age (*F*(1,78) = 35.260, *p* < .001, η_p_^2^ = .311). There was also no interaction between context and age (*F*(2,156) = 2.051, *p* = .132, η_p_^2^ = .026). Looking at effects of task demands, there was no main effect of task demand (*F*(1,78) = 2.298, *p* = .134, η_p_^2^ = .029), suggesting that reading times were not affected by answering questions versus simply reading (see Table 3).

There was also no significant interaction between age group and task demands (*F*(1, 78) = .259, *p* = .612, η_p_^2^ = .003), which suggests that the overall reading time difference between younger and older adults remained the same when answering questions or simply reading. In other words, neither younger nor older adults’ reading times were influenced by task demands. There was also no significant interaction between context and task demands (*F*(2,156) = .759, *p* = .470, η_p_^2^ = .010), which suggests that the match effect did not differ when either answering questions or simply reading (see Table 3 and Fig 1).

The analysis found no significant three-way interaction between age group, sentence, context, and task demands. This suggests that the facilitating match effect observed for younger and older adults did not differ when either answering questions or reading for comprehension (*F*(2, 156) = 2.367, *p* = .097, η_p_^2^ = .029; see Table 3 and Fig 1).

### 3.3. NASA

Participants completed a NASA rating after the self-paced reading task with questions and after the self-paced reading task without questions. An overall NASA was also completed at the end of the entire reading task so that participants could rate their overall experience of workload. An independent t-test was computed to determine whether there was a significant difference between younger and older adults in terms of overall workload. The overall NASA (Table 4) score showed no significant difference between younger and older adults in terms of overall workload experience (*t*(78) = -.482, *p* = .631, *d* = .108). This suggested that both older and younger adults experienced a similar level of workload when completing the tasks. When a mixed ANOVA was conducted to compare the parts with and without questions, there was no main effect of age either (*F*(1, 78) = .291, *p* = .591, η_p_^2^ = .004) and there was no significant effect of task demand (*F*(1, 78) = .318, *p* = .575, η_p_^2^ = .004), suggesting that the experience of workload did not significantly differ between the two tasks. There was no significant interaction between age and task demand (*F*(1, 78) = .933, *p* = .337, η_p_^2^ = .012), reflecting that neither age group showed a workload effect.

**Table 4:** Average NASA score (0–100) and standard deviations for each task in each age group.

	Younger adults	Older adults
**NASA**		
*Questions*	52.09 (16.17)	51.10 (16.12)
*No questions*	48.31 (16.35)	52.09 (15.39)
**Overall NASA**	50.40 (16.76)	52.10 (14.81)

## 4. Discussion

The aim of this study was to assess whether age influences word processing during language comprehension and to determine whether sentence contexts and task demands modulate age effects further. To examine the effect of sentence context, three different contexts were used (matched, mismatched, and neutral) during a self-paced reading task to measure the effect context had on reading times. To examine the effect of task demand, participants either read the sentences for comprehension only or answered a question after each sentence to measure the effect this had on reading times.

Average scores from the likeliness-rating task confirmed that target words were most likely to be found in matching contexts and least likely in mismatched contexts. The results from the self-paced reading task showed that matched sentence contexts facilitated reading times for both younger and older adults. This matching effect did not differ between younger and older adults. The results from the self-paced reading task thus suggest that both younger and older adults can benefit from context predicting upcoming words. This was found both when simply reading and when reading to answer questions, with task demands not influencing reading times. We also hypothesised that there would be a mismatch effect, which was not observed. Mismatched sentence contexts did not hinder sentence processing and showed similar reading times as neutral contexts for older or younger adults. This suggests that both older and younger adults were able to benefit from semantic knowledge predicting upcoming words while neither age group experienced hindrance from contexts mismatching the target word.

### 4.1. Matched sentence contexts and ageing

Previous studies assessing effects of matched, constraining contexts on language comprehension in younger and older adults have shown mixed findings. In general, studies suggest that older adults can benefit from context predicting or priming upcoming words. However, while some studies suggest that older adults can benefit from semantic context to the same extent as younger adults [10, 22–24] or even more than younger adults [20, 21], other studies have suggested that older adults cannot use the semantic context as effectively [6–8].

Our findings are in line with research indicating that older adults are facilitated by matched contexts to the same extent as younger adults (e.g., [10, 22–24]). This supports the finding that older adults do have intact semantic knowledge, which they can use to facilitate their reading times as well as younger adults during language comprehension [13–15]. During comprehension, the first words in the matching sentence semantically primed upcoming words (i.e., the target). This priming might have increased activation of the target words, which consequently might have been processed faster when encountered later in the sentence. In addition, the matching context might have allowed both younger and older adults to form predictions about the upcoming target words based on the preceding context, which in turn might have sped up reading times.

While studies using a range of paradigms (e.g., reading times, ERP, and eye-tracking data) have shown processing differences between constraining sentences and neutral or no contexts, or between expected and unexpected words, the age effects are more mixed. Across the literature, studies using behavioural measures such as reading times seem more likely to find that older adults use sentence context (at least) as much as younger adults. ERP studies, in contrast, more often suggest that older adults’ processing is less influenced by sentence context. Recent research [33] has suggested that this might be because reading times and ERP components are influenced differently by lexical versus semantic predictions. At a lexical level, both “salt” and “sock” would be unexpected in a sentence starting with “I take my coffee with cream and …”, while “sugar” would be the predicted lexical word. In terms of semantic predictions, however, “salt” shares semantic features with “sugar” (i.e., both are edible and white). Listeners might be predicting words by using lexical predictions (i.e., which word forms are likely) as well as semantic predictions (i.e., what type of semantic features are likely). Older adults might use similar lexical predictions as younger adults [33]. This type (lexical predictions) might furthermore be the type of predictions that are most likely to influence word reading times. If older adults’ lexical predictions are indeed intact, paradigms assessing reading times should be more likely to observe similar performance in older and younger adults. Our study further supports the argument that older adults can indeed use lexical predictions to the same extent as younger adults. In contrast, N400 effects in ERP studies might be more sensitive to both lexical and semantic predictions. The latter type (semantic predictions) is argued to change more with age [33]. ERP studies might therefore be more likely to show smaller context effects in older adults. Differences across studies might be further influenced by the type of comparisons made, especially when constraining sentences with expected words are compared to unexpected words (as opposed to our comparison between constraining, matching sentences and neutral contexts).

### 4.2. Mismatched sentence contexts and ageing

We also compared neutral and mismatched sentence contexts to assess how well older and younger adults could inhibit interfering information. A mismatch effect was not observed: neither younger nor older adults’ reading times were affected by mismatching sentence contexts. This suggests interference from these contexts was not sufficient to hinder their comprehension. These findings appear to go against research suggesting that older adults have diminished semantic or inhibitory control [13, 16]. However, given that the mismatch effect was not observed at all in either age group, one possible explanation is that the words presented in the mismatching sentences used in our study were not interfering enough with the target word. Previous studies assessing mismatching sentence contexts in younger adults have often used stronger semantic violations (e.g., drinking coffee with cream and socks). In our study, mismatched sentences were not semantic anomalies. Although they were less plausible, most sentences did not strongly violate semantic or world knowledge. This ensured that the study assessed interference of irrelevant information rather than interference of impossible words. As a result, however, effects of interference might have been smaller (or absent) than in contexts used in previous studies that looked at stronger violations of world or semantic knowledge (e.g., [25]). Indeed, previous studies have observed the strongest effect on processing when a word is unexpected and unrelated (e.g., “coffee with cream and *socks*”; e.g., [6, 34]). However, studies have also shown that processing can still be influenced when possible but unlikely target words are presented (e.g., Dutch trains being white instead of yellow [12] or sentences like “coffee with cream and *salt*” [33]). It is therefore possible that interference from mismatching contexts was not entirely absent in our study but rather that it was too small to influence reading times. It is possible that both younger and older adults were able to suppress the (small amount of) interference stemming from mismatched contexts within the same amount of time they needed to process words presented in neutral contexts. Finally, future research is needed to examine whether mismatch effects might build up with time. As expected, reading became faster when the target word was repeated (e.g., [35]). Older adults benefited more from word repetition than younger adults [36]. This exploratory analysis assessing effects of target-word repetition showed an influence of repetition on reading times in all conditions. However, when comparing the third versus second presentation, reading times in the match and neutral conditions continued to decrease, but this was no longer the case for mismatch trials. When a target word’s third repetition was accompanied by a mismatching sentence context, that same word had already been presented with a neutral and matching context. It is possible that earlier presentations of the same word in a fitting context increased the mismatch response to these words. Thus, mismatch effects might perhaps be more likely to occur when stronger context expectations are created for each target word first. However, given the low number of trials remaining per context when examining the three target-word repetitions, these patterns should be interpreted with great caution.

### 4.3. Task demands

Task demand was an exploratory variable within the current study to assess if age effects were modulated by potential increases in cognitive load as a consequence of asking questions after each sentence. The results suggested that neither age effects nor context effects were modulated by task demand, as reading times did not increase for any age group or context when answering additional questions as compared to reading for comprehension only. Furthermore, workload experience was comparable for the tasks with and without questions, and for the two age groups. This suggests that participants did not find the part with questions more difficult to complete, and consequently reading times were unaffected. It is, however, possible that both task conditions (with and without questions) posed relatively low demands. Self-paced reading tasks give participants time to process and integrate words and to form predictions [8]. Tasks that provide participants with less time (e.g., listening to natural speech) might increase task demands further than reading tasks and as such might be more likely to show age-group differences.

### 4.4. Limitations

One limitation of this research is that we observed a larger overall RT difference in reading times between age groups than would perhaps be expected [e.g., 27]. Since participants completed the study online without being monitored, this large age effect could be due to a difference in the age groups’ intentions for completing the study, rather than a difference in their processing or reading. Younger adults might be participating to earn money, whereas older adults might participate out of interest or entertainment. As a result, younger participants may rush the study while older adults might be more inclined the complete the study correctly [37]. However, in the current study, accuracy when answering the comprehension questions was high for all participants. This suggests that even if participants were going through the task quickly, this did not negatively affect their performance in terms of language comprehension. Furthermore, RTs did not differ for the parts with and without questions, suggesting that participants did not read the sentences more carefully when they had to answer questions (i.e., it was not the case that they only paid attention when questions were asked). Workload experience was also similar for the two age groups. In addition, similar results (with no interaction between context and age group) were observed when we z-scored the RTs to consider differences between age groups in overall RTs.

## 5. Conclusion

The overall findings suggest that the reading times of younger and older adults are equally facilitated by matched sentence contexts, as their semantic knowledge enables initial words to semantically prime, or predict, upcoming words for quicker processing upon exposure. This match effect was not modulated by task demand for either age group, suggesting that the ability to comprehend language is impacted by the formulation of a sentence rather than the level of difficulty of the task in which the sentence is found. In contrast, a mismatch effect was not observed, indicating that neither younger nor older adults were hindered by mismatched contexts, as they might be able to inhibit (weakly) interfering information to the same extent during comprehension. These results have positive implications as it suggests that facilitating sentence contexts can be used to help word processing during comprehension in older adults by semantically priming words for improved recognition. This research also suggests that mismatching sentence contexts do not always hinder younger or older adults in their ability to comprehend language.

## Supporting information

S1 File The supporting information contains all sentences and questions used in the reading experiment. (DOCX)
