# Supplementary material for: Diving into a pool or volcano? Examining the influence of sentence context and task demands on sentence reading in younger and older adults
Source: PLoS One. 2022 Dec 30;17(12):e0279555. doi: 10.1371/journal.pone.0279555 (PMC9803221; doi:10.1371/journal.pone.0279555)
Supplement: S1 File — (DOCX) [file pone.0279555.s001.docx]

**Supporting Information**

*Table S1.* Sentences used in the self-paced reading task. Each triplet of sentences was presented with one of the two corresponding targets. This way, half of the participants saw a given sentence in the matched context (e.g., “the girl wrapped **the present**”) while the other half of the participants saw the same sentence in the mismatched context (e.g., “the girl wrapped **the leg**”).

| **SENTENCES** | | | |
| --- | --- | --- | --- |
| **Target** | **Matched** | **Mismatched** | **Neutral** |
| Present  *Leg* | The girl wrapped the present before Christmas.  *The footballer broke his leg at the match.* | The footballer broke his present at the match.  *The girl wrapped the leg before Christmas.* | The grandpa perceived the present on Tuesday.  *The grandpa perceived the leg on Tuesday.* |
| Potato  *Banana* | The dinner lady mashed the potato before tea.  *The monkey ate the banana in the trees.* | The monkey ate the potato in the trees.  *The dinner lady mashed the banana before tea.* | The cat spent his time examining the potato on the floor.  *The cat spent his time examining the banana on the floor.* |
| Knife  *Bone* | The defendant was accused of stabbing his victim with the knife in an alleyway.  *The dog licked the bone that his owner gave to him.* | The dog licked the knife that his owner gave to him.    *The defendant was accused of stabbing his victim with a bone in an alleyway.* | The young man was carrying the knife in his bag.  *The young man was carrying the bone in his bag.* |
| Ring  *Crown* | Married women wear a ring on their finger.  *The queen wore a crown on her coronation day.* | The queen wore the ring on her coronation day.  *Married women wear a crown on their finger.* | The girl lifted the ring off the table and smiled.  *The girl lifted the crown off the table and smiled.* |
| Bible  *Fairytale* | The priest read the bible at the mass.  *Before bed the father read the fairytale to his daughter.* | Before bed the father read the bible to his daughter.  *The priest read the fairytale at the mass.* | The young man picked up the bible from the shelf.  *The young man picked up the bible from the shelf.* |
| Lamp  *Oven* | The woman went to bed and turned off the lamp before falling asleep.  *The chef switched on the oven before preparing the meal.* | The chef switched on the lamp before preparing the meal.  *The woman went to bed and turned off the oven before falling asleep.* | The man used the lamp during the day.  *The man used the oven during the day.* |
| Roses  *Money* | The man gave his date roses before they ate dinner.  *The thief stole money from the bank.* | The thief stole roses from the bank.  *The man gave his date money before they ate dinner.* | The girl requested roses from the shop keeper.  *The girl requested money from the shop keeper.* |
| Bin  *Bucket* | The cleaner threw away rubbish into the bin during their shift.  *The little girl put sand in the bucket when she went to the beach.* | The little girl put sand in the bin when she went to the beach.  *The cleaner threw away rubbish into the bucket during their shift.* | The children cleaned the bin as punishment.  *The children cleaned the bucket as punishment.* |
| Ice  *Butter* | The customer ordered whiskey with ice at the pub.  *The pastry chef melted butter to make bread.* | The pastry chef melted ice to make bread.  *The customer ordered whiskey with butter at the pub.* | The student chilled the ice in the fridge.  *The student chilled butter in the fridge.* |
| Ship  *Aeroplane* | The captain navigated the ship back to the harbour.  *The pilot crashed the aeroplane into a building.* | The pilot crashed the ship into a building.  *The captain navigated the aeroplane back to the harbour.* | The character destroyed the ship with a hammer.  *The character destroyed the aeroplane with a hammer.* |
| Duck  *Baby* | The toddler fed bread to the duck near the pond.  *The new mum was feeding her baby in her living room.* | The new mum was feeding her duck in her living room.  *The toddler fed bread to the baby near the pond.* | The little girl was distracted from her toys by the duck that was nearby.  *The little girl was distracted from her toys by the baby that was nearby.* |
| Bottle  *Flask* | The hikers drank water from a bottle during the expedition.  *The commuter drank coffee from a flask on his way to work.* | The commuter drank coffee from a bottle on his way to work.  *The hikers drank water from a flask during the expedition.* | The guy swiftly bought the bottle that was for sale.  *The guy swiftly bought the flask that was for sale.* |
| Fireworks  *Candles* | The dog was frightened of fireworks on bonfire night.  *The lady’s dining table was lit with candles for her evening meal.* | The lady's dining table was lit with fireworks for her evening meal.  *The dog was frightened of candles on bonfire night.* | They decided to put the fireworks into a box.  *They decided to put the candles into a box.* |
| Bread  *Cake* | The baker used yeast to make bread for his bakery.  *The birthday girl ate cake with her family.* | The birthday girl ate bread with her family.  *The baker used yeast to make cake for his bakery.* | The pupil made the bread in his spare time.  *The pupil made the cake in his spare time.* |
| Mountain  *Ladder* | The explorer climbed the mountain in the Alps.  *The construction worker climbed a ladder to fix a cracked pipe.* | The construction worker climbed a mountain to fix a cracked pipe.  *The explorer climbed the ladder in the Alps.* | They finally reached the mountain and took a break.  *They finally reached the ladder and took a break.* |
| Bandage  *Blanket* | The injured man wrapped the bandage around the wound.  *The midwife wrapped the newborn in the blanket at the hospital.* | The midwife wrapped the newborn in the bandage at the hospital.  *The injured man wrapped the blanket around the wound.* | The consumer grabbed the bandage from the man.  *The consumer grabbed the blanket from the man.* |
| Horse  *Bike* | The jockey rode the horse at the races.  *The mail boy travelled by bike to deliver the mail.* | The mail boy travelled by horse to deliver the mail.  *The jockey rode the bike at the races.* | The competitor lost the horse during the challenge.  *The competitor lost the bike during the challenge.* |
| Calendar  *Statue* | The mother scheduled family events on the calendar so she did not forget.  *The sculptor created the statue for the art exhibition.* | The sculptor created the calendar for the art exhibition.  *The mother scheduled family events on the statue so she did not forget.* | The lady created the calendar as a birthday present for her friend.  *The lady created the statue as a birthday present for her friend.* |
| Hair  *Meat* | The barber cut the hair into a modern hairstyle.  *The butcher chopped the meat for his customer.* | The butcher chopped the hair for his customer.  *The barber cut the meat into a modern hairstyle.* | She closely inspected the hair for several minutes.  *She closely inspected the meat for several minutes.* |
| Carrot  *Cheese* | The rabbit ate the carrot for breakfast.  *The mouse nibbled on the cheese when it was hungry.* | The mouse nibbled on the carrot when it was hungry.  *The rabbit ate the cheese for breakfast.* | The associates purchased the carrot for their endeavour.  *The associates purchased the cheese for their endeavour.* |
| Purse  *Jar* | She carried money in her purse when she went shopping.  *She stored cookies in the jar once she had opened the packet.* | She stored the cookies in the purse once she had opened the packet.  *She carried money in her jar when she went shopping.* | The passerby held the lady's purse in his hand.  *The passerby held the lady’s jar in his hand.* |
| Painting  *Dress* | The artist admired the painting in the art gallery.  *The tailor altered the dress for her client.* | The tailor altered the painting for her client.  *The artist admired the dress in the art gallery.* | The viewer commented on the painting at the event.  *The viewer commented on the dress at the event.* |
| Bag  *Cot* | The athlete kept his kit in the bag at the gym.  *The babysitter laid the sleeping baby into the cot while she drank her tea*. | The babysitter laid the sleeping baby into the bag while she drank her tea.  *The athlete kept his kit in the cot at the gym.* | The guest fixed the bag at the weekend.  *The guest fixed the cot at the weekend.* |
| Bed  *Car* | The granny slept in bed most days when she was ill.  *The automobile salesman sold the car to his customer.* | The automobile salesman sold the bed to his customer.  *The granny slept in the car most days when she was ill.* | The visitor purchased the bed at the establishment and returned home.  *The visitor purchased the car at the establishment and returned home.* |
| Rattle  *Egg* | The baby shook the rattle on his play mat.  *The chef cracked the egg on the floor.* | The chef cracked the rattle on the floor.  *The baby shook the egg on his play mat.* | The father asked his son to bring the rattle to the park.  *The father asked his son to bring the egg to the park.* |
| Bank  *Nursery* | The thief robbed the bank over the summer holidays.  *The mum dropped her young daughter off at the nursery before work.* | The mum dropped her young daughter off at the bank before work.  *The thief robbed the nursery over the summer holidays.* | The man called the bank to discuss a query.  *The man called the nursery to discuss a query.* |
| Beer  *Tea* | The student went to the bar to buy a beer for her friend.  *The grandma sweetened the tea for her grandson.* | The grandma sweetened the beer for her grandson.  *The student went to the bar to buy a tea for her friend.* | The grandpa discarded the beer before leaving.  *The grandpa discarded the tea before leaving.* |
| Ball  *Treasure* | The school children played with the ball at break time.  *The pirate discovered the treasure in the treasure chest.* | The pirate discovered the ball in the treasure chest.  *The school children played with the treasure at break time.* | The group decided to share the ball after their hard work paid off.  *The group decided to share the treasure after their hard work paid off.* |
| Tree  *Door* | The squirrel climbed the tree to escape the barking dog.  *The security guard stood next to the door outside the club.* | The security guard stood next to the tree outside the club.  *The squirrel climbed the door to escape the barking dog.* | The committee removed the tree after a long debate.  *The committee removed the door after a long debate.* |
| Rope  *Laces* | The sailor knotted the rope tightly to the boat.  *The little boy was learning to tie the laces on his new trainers.* | The little boy was learning to tie the rope on his new trainers.  *The sailor knotted the laces tightly to the boat.* | The team forgot to put the rope in the bag before setting off.  *The team forgot to put the laces in the bag before setting off.* |
| Wood  *Nails* | The carpenter cut the wood to make a bench.  *The beautician filed the nails of her client.* | The beautician filed the wood of her client.  *The carpenter cut the nails to make a bench.* | The girl observed the wood being stolen.  *The girl observed the nails being stolen.* |
| Glue  *Bubblegum* | The teacher stuck the paper together with glue to make a cube.  *The kid chewed the bubblegum in the corner of the classroom.* | The kid chewed glue in the corner of the classroom.  *The teacher stuck the paper together with bubblegum to make a cube.* | The teenager retrieved the glue from the box in the cupboard.  *The teenager retrieved the bubblegum from a box in the cupboard.* |
| Finger  *Plant* | He put the ring on her finger after she had said yes.  *The gardener cut the plant with his hedge cutters.* | The gardener cut the finger with his hedge cutters.  *He out the ring on her plant after she had said yes.* | The new occupant found the finger in the living room.  *The new occupant found the plant in the living room.* |
| Chimney  *Floor* | The smoke was billowing out of the chimney at the flat.  *The maid swept the floor in the kitchen.* | The maid swept the chimney in the kitchen.  *The smoke was billowing out of the floor at the flat.* | The guardian assessed the chimney for problems.  *The guardian assessed the floor for problems.* |
| Prisoner  *Patient* | The jailer escorted the prisoner back to his cell.  *The doctor treated the patient for arthritis.* | The doctor treated the prisoner for arthritis.  *The jailer escorted the patient back to his cell.* | The spectator secretly met the prisoner before the meeting.  *The spectator secretly met the patient before the meeting.* |
| Gardener  *Policewoman* | The hedge was trimmed by the gardener into the shape of a rooster.  *The resident alerted the policewoman of a security breach.* | The resident alerted the gardener of a security breach.  *The hedge was trimmed by the policewoman into the shape of a rooster.* | The candidate consulted the gardener for advice.  *The candidate consulted the policewoman for advice.* |
| Tin  *Bowl* | The baked beans were stored in the tin in a large cupboard.  *The caterer ladled soup into the bowl for their guests.* | The caterer ladled soup into the tin for their guests.  *The baked beans were stored in the bowl in a large cupboard.* | The parents promptly handed over the tin to the supervisor.  *The parents promptly handed over the bowl to the supervisor.* |
| Hand  *Hook* | The candidate shook the employer's hand at the interview.  *The host hung the coat on the hook in the cloak room.* | The host hung the coat on the hand in the cloak room.  *The candidate shook the employer’s hook at the interview.* | The man touched the hand before leaving.  *The man touched the hook before leaving.* |
| Shuttlecock  *Brush* | The badminton player hit the shuttlecock and scored three points.  *The stablehand groomed the horse with the brush before the dressage show.* | The stablehand groomed the horse with the shuttlecock before the dressage show.  *The badminton player hit the brush and scored three points.* | The trainees received the shuttlecock at the training session.  *The trainees received a brush at the training session.* |
| Mobile  *Trowel* | The patient rang the doctor holding their mobile to book an appointment.  *The bricklayer used the trowel to lay the cement.* | The bricklayer used the mobile to lay the cement.  *The patient rang the doctor holding their trowel to book an appointment.* | The employee was immediately given the mobile to use at work.  *The employee was immediately given the trowel to use at work.* |
| Water  *Jam* | The waiter spilled the jug of water on the dining table.  *The granny used strawberries to make jam for her jam tarts.* | The granny used strawberries to make water for her jam tarts.  *The waiter spilled the jug of jam on the dining table.* | The four campers had taken lots of water on their camping trip.  *The four campers had taken lots of jam on their camping trip.* |
| Ribbon  *Lock* | The girl tied her hair with the ribbon into a ponytail.  *The cyclist secured his bike with the lock at the bike rack.* | The cyclist secured his bike with the ribbon at the bike rack.  *The girl tied her hair with the lock into a ponytail.* | The students were given a ribbon in their art class for inspiration.  *The students were given a lock in their art class for inspiration.* |
| Shirt  *Poster* | The maid ironed the shirt before washing the dishes.  *The advertisers stuck the poster onto the wall.* | The advertisers stuck the shirt onto the wall.  *The maid ironed the poster before washing the dishes.* | The gentleman was handed a shirt from the pile.  *The gentleman was handed a poster from the pile.* |
| Letter  *Medicine* | The woman posted the letter into the letterbox.  *The nurse gave the patient their medicine before their checkup.* | The nurse gave the patient their letter before their checkup.  *The woman posted the medicine into the letterbox.* | The customer requested the letter at the counter.  *The customer requested the medicine at the counter.* |
| Cell  *Hotel* | The criminal was locked up in the cell at night.  *During the holiday the couple stayed in the hotel at night.* | During the holiday the couple stayed in the cell at night.  *The criminal was locked up in the hotel at night.* | The couple visited the cell at the beginning of the evening.  *The couple visited the hotel at the beginning of the evening.* |
| Lightning  *Sun* | During the thunderstorm they saw lightning from their bedroom window.  *The girls got a golden tan from the sun in summer.* | The girls got a golden tan from the lightning in summer.  *During the thunderstorm they saw sun from their bedroom window.* | In the morning they woke up to lightning outside.    *In the morning they woke up to sun outside.* |
| Envelope  *Nest* | He put a stamp on the envelope and posted it.  *The birds lay their eggs in the nest each spring.* | The birds lay their eggs in the envelope each spring.  *He put a stamp on the nest and posted it.* | They picked up the envelope on the floor.  *They picked up the nest on the floor.* |
| Umbrella  *Watch* | During the rainstorm he used the umbrella for shelter.  *He checked the time on his watch before the meeting.* | He checked the time on his umbrella before the meeting.  *During the rainstorm he used the watch for shelter.* | The brother took the umbrella with him all the time.  *The brother took the watch with him all the time.* |
| Pasta  *Soil* | The Italian restaurant serves pasta and pizza.  *He put the flowers in soil to keep them alive.* | He put the flowers in pasta to keep them alive.  *The Italian restaurant serves soil and pizza.* | The young lady suddenly threw the pasta in the air.  *The young lady suddenly threw soil in the air.* |
| Pool  *Volcano* | The swimmer dived into the pool and began training.  *The man watched the lava erupt from the volcano with dismay.* | The man watched the lava erupt from the pool with dismay.  *The swimmer dived into the volcano and began training.* | They went to see the pool in the evening with their friends.  *They went to see the volcano in the evening with their friends.* |
| Axe  *Stick* | The lumberjack required the axe to cut down the tree.  *The dog enjoyed chasing the stick in the park.* | The dog enjoyed chasing the axe in the park.  *The lumberjack required* *the stick to cut down the tree.* | The cat watched the man with the axe walking down the street.  *The cat watched the man with the stick walking down the street.* |
| Gun  *Stapler* | The criminal reloaded the gun within seconds.  *The receptionist bound the paper with the stapler for filing.* | The receptionist bound the paper with the gun for filing.  *The criminal reloaded the stapler within seconds.* | The technician slowly fixed the gun for the gentleman.  *The technician slowly* *fixed the stapler for the gentleman.* |
| Potion  *Rubbish* | The witch stirred the potion in her cauldron.  *The dustman disposed of the rubbish in the morning.* | The dustman disposed of the potion in the morning.  *The witch stirred the rubbish in her cauldron.* | The boy made use of the potion in a clever way.  *The boy made use of the rubbish in a clever way.* |
| Artist  *Tenant* | The sketch was drawn by the artist at the fair.  *The landlord evicted the tenant with very short notice.* | The landlord evicted the artist with very short notice.  *The sketch was drawn by the tenant at the fair.* | The gang of youths made fun of the artist and laughed.  *The gang of youths made fun of the tenant and laughed.* |
| Table  *Bed* | At teatime the family sat at the table to eat.  *The man bought a mattress for the bed last weekend.* | The man bought a mattress for the table last weekend.  *At teatime the family sat at the bed to eat.* | The culprit damaged the table during the night.  *The culprit damaged the bed during the night.* |
| Wardrobe  *Mirror* | She hung up her clothes in the wardrobe after work.  *She looked at her reflection in the mirror before going out.* | She looked at her reflection in the wardrobe before going out.  *She hung up her clothes in the mirror after work.* | The cleaner thoroughly washed the wardrobe once a week.  *The cleaner thoroughly washed the mirror once a week.* |
| Trainers  *Gloves* | The runner wore a new pair of trainers for the race.  *The eskimo wore a pair of gloves in the snowy weather.* | The eskimo wore a pair of trainers in the snowy weather.  *The runner wore a new pair of gloves for the race*. | The child hid the pair of trainers in the drawer.  *The child hid the pair of gloves in the drawer.* |
| Castle  *Cave* | The king lived in the castle with the queen.  *The dragon lived in the cave his entire life*. | The dragon lived in the castle his entire life.  *The king lived in the cave with the queen.* | The police found the suspect in the castle years later.  *The police found the suspect in the cave years later*. |
| Cow  *Hamster* | The farmer milked the cow early in the morning.  *The parents went to the petshop to buy the hamster for their son.* | The parents went to the petshop to buy the cow for their son.  *The farmer milked the hamster early in the morning.* | They took a picture of the cow on their day out.  *They took a picture of the hamster on their day out.* |
| Notepad  *Rucksack* | The student wrote bullet points in the notepad during the lecture.  *The children kept their lunch in the rucksack until lunchtime.* | The children kept their lunch in the notepad until lunchtime.  *The students wrote bullet points in the rucksack during the lecture.* | During the day the driver kept the notepad in the boot of his car.  *During the day the driver kept the rucksack in the book of his car.* |

*Table S2.* Questions used in the condition with questions. There was an equal number of questions asking about the context or the target word of the sentence in each condition. Sentences in bold asked about the target. Each condition included who/what questions for approximately two-thirds of the sentences and when/where questions for one-thirds of the sentences.

| **QUESTIONS** | | | |
| --- | --- | --- | --- |
| **Target** | **Matched** | **Mismatched** | **Neutral** |
| *Present*  *Leg* | Who wrapped the present?  Who broke his leg at the match? | Who broke his present at the match?  Who wrapped the leg? | When did grandpa perceive the present?  When did grandpa perceive the leg? |
| *Potato*  *Banana* | When did the dinner lady mash the potato?  Where did the monkey eat the banana? | Where did the monkey eat the potato?  When did the dinner lady mash the banana? | Who examined the potato?  Who examined the banana? |
| *Knife*  *Bone* | Where was the defendant accused of stabbing his victim?  Who gave the dog the bone? | Who gave the dog the knife?  Where was the defendant accused of stabbing his victim? | Where did the man carry the knife?  Where did the man carry the bone? |
| *Ring*  *Crown* | Who wears a ring on their finger?  When did the queen wear a crown? | When did the queen wear a ring?  Who wears a crown on their finger? | Who lifted the ring off the table?  Who lifted the crown off the table? |
| *Bible*  *Fairytale* | Who read the bible at the mass?  Who read the fairytale to the daughter? | Who read the bible to the daughter?  Who read the fairytale at the mass? | Where did the man pick up the bible from?  Where did the man pick up the fairytale from? |
| *Lamp*  *Oven* | **What did the woman turn off before bed?**  **What did the chef switch on?** | **What did the chef switch on?**  **What did the woman turn off before bed?** | When did the man use the lamp?  When did the man use the oven? |
| *Roses*  *Money* | **What did the man give to his date?**  **What did the thief steal from the bank?** | **What did the thief steal from the bank?**  **What did the man give to his date?** | Who did the girl request roses from?  Who did the girl request money from? |
| *Bin*  *Bucket* | What did the cleaner throw away?  Who put sand in the bucket? | Who put sand in the bin?  What did the cleaner throw away? | What did the children do to the bin?  What did the children do to the bucket? |
| *Ice*  *Butter* | Where did the customer order whiskey?  What did the pastry chef make? | What did the pastry chef make?  Where did the customer order whiskey? | Who chilled the ice?  Who chilled the butter? |
| *Ship*  *Aeroplane* | Where did the captain navigate the ship?  What did the pilot crash the aeroplane into? | What did the pilot crash the ship into?  Where did the captain navigate the pilot? | What did the character destroy the ship with?  What did the character destroy the aeroplane with? |
| *Duck*  *Baby* | Who fed the duck?  Where was the mum feeding the baby? | Where was the mum feeding the duck?  Who fed the baby? | Where was the duck?  Where was the baby? |
| *Bottle*  *Flask* | What did the hikers do during the expedition?  Who drank coffee from a flask? | Who drank coffee from a bottle?  What did the hikers do during the expedition? | Who bought the bottle?  Who bought the flask? |
| *Fireworks*  *Candles* | When was the dog frightened?  Whose table was lit with candles? | Whose table was lit with fireworks?  When was the dog frightened? | Where did they put the fireworks?  Where did they put the candles? |
| *Bread*  *Cake* | What did the baker use to make bread?  Who did the birthday girl eat with? | Who did the birthday girl eat with?  What did the baker use to make cake? | Who made bread in their spare time?  Who made cake in their spare time? |
| *Mountain*  *Ladder* | Where did the explorer climb the mountain?  What did the construction worker fix? | What did the construction worker fix?  Where did the explorer climb the ladder? | **Where did they take a break?**  **Where did they take a break?** |
| *Bandage*  *Blanket* | Who wrapped the bandage around the wound?  Who wrapped the newborn in a blanket? | Who wrapped the newborn in a bandage?  Who wrapped the blanket around the wound? | Who grabbed the bandage from the man?  Who grabbed the blanket from the man? |
| *Horse*  *Bike* | Where was the horse ridden?  **How did the mailboy travel?** | **How did the mailboy travel?**  Where was the horse ridden? | Who lost the horse?  Who lost the bike? |
| *Calendar*  *Sculptor* | What was scheduled on a calendar?  **What did the sculptor create?** | **What did the sculptor create?**  What was scheduled on a statue? | What was the calendar for?  What was the statue for? |
| *Hair*  *Meat* | What type of hairstyle did the barber cut?  Who chopped the customer’s meat? | Who chopped the customer’s hair?  What type of hairstyle did the barber cut? | How long did she inspect the hair for?  How long did she inspect the meat for? |
| *Carrot*  *Cheese* | Who ate the carrot for breakfast?  Who nibbled on the cheese? | Who nibbled on the carrot?  Who ate the cheese for breakfast? | Who purchased the carrot?  Who purchased the cheese? |
| *Purse*  *Jar* | Where did she carry money in her purse?  What did she store in her jar? | What did she store in her purse?  Where did she carry money in her jar? | Who held the lady’s purse?  Who held the lady’s jar? |
| *Painting*  *Dress* | **What did the artist admire?**  **What did the tailor alter?** | **What did the tailor alter?**  **What did the artist admire?** | Where did the viewer comment on the painting?  Where did the viewer comment on the dress? |
| *Bag*  *Cot* | **Where did the athlete keep his kit?**  Who laid the baby into a cot? | Who laid the baby into a bag?  **Where did the athlete keep his kit?** | When did the guest fix the bag?  When did the guest fix the cot? |
| *Bed*  *Car* | Who slept in bed most days when they were ill?  What type of salesman sold a car to his customer? | What type of salesman sold a bed to his customer?  Who slept in the car most days when they were ill? | Where did the visitor purchase the bed?  Where did the visitor purchase the car? |
| *Rattle*  *Egg* | Where did the baby shake the rattle?  Where did the chef crack the egg? | Where did the chef crack the rattle?  Where did the baby shake the egg? | **What did the father ask his son to bring?**  **What did the father ask his son to bring?** |
| *Bank*  *Nursery* | When did the thief rob the bank?  Who did the mum drop off at the nursery? | Who did the mum drop off at the bank?  When did the thief rob the nursery? | **Who did the man call?**  **Who did the man call?** |
| *Beer*  *Tea* | Who did the student buy beer for?  Who did the grandma sweeten the tea for? | Who did the grandma sweeten the beer for?  Who did the student buy tea for? | **What did the grandpa discard?**  **What did the grandpa discard?** |
| *Ball*  *Treasure* | When did the school children play with the ball?  Where did the pirate discover the treasure? | Where did the pirate discover the ball?  When did the school children play with the treasure? | Who shared the ball?  Who shared the treasure? |
| *Tree*  *Door* | What did the squirrel escape from?  Who was stood next to the door? | Who was stood next to the tree?  What did the squirrel escape from? | **What did the committee remove?**  **What did the committee remove?** |
| *Rope*  *Laces* | **What did the sailor knot tightly to the boat?**  **What was the little boy learning to tie?** | **What was the little boy learning to tie?**  **What did the sailor knot tightly to the boat?** | Who forgot to put the rope in the bag?  Who forgot to put the laces in the bag? |
| *Wood*  *Nails* | What did the carpenter cut wood to make?  Who filed the nails? | Who filed the wood?  What did the carpenter cut nails to make? | Who observed the wood being stolen?  Who observed the nails being stolen? |
| *Glue*  *Bubblegum* | What did the teacher make out of paper?    Where did the kid chew bubblegum? | Where did the kid chew glue?  What did the teacher make out of paper? | Where did the teenager retrieve the glue from?  Where did the teenager retrieve the bubblegum from? |
| *Finger*  *Plant* | **Where did he put the ring?**  What did the gardener cut his plant with? | What did the gardener cut his finger with?  **Where did he put the ring?** | **What did the new occupant find?**  **What did the new occupant find?** |
| *Chimney*  *Floor* | Where was smoke billowing from the chimney?  Where was the floor that the maid swept? | Where was the chimney that the maid swept?  Where was smoke billowing from the floor? | Who assessed the chimney?  Who assessed the floor? |
| *Prisoner*  *Patient* | Where did the jailer escort the prisoner to?  What did the doctor treat the patient for? | What did the doctor treat the prisoner for?  Where did the jailer escort the patient to? | **Who did the spectator meet?**  **Who did the spectator meet?** |
| *Gardener*  *Policewoman* | **Who trimmed the hedge?**  Who alerted the policewoman? | Who alerted the policewoman?  **Who trimmed the hedge?** | **Who did the candidate consult?**  **Who did the candidate consult?** |
| *Tin*  *Bowl* | **What were the baked beans stored in?**  **What did the caterer ladle the soup into?** | **What did the caterer ladle soup into?**  **What were the baked beans store in?** | Who did the parents hand the tin to?  Who did the parents hand the bowl to? |
| *Hand*  *Hook* | Who shook the employer’s hand?  **What did the host hang the coat on?** | **What did the host hang the coat on?**  Who shook the employer’s hook? | **What did the man touch before leaving?**  **What did the man touch before leaving?** |
| *Shuttlecock*  *Brush* | **What was hit?**  **What did the stablehand groom the horse with?** | **What did the stablehand groom the horse with?**  **What was hit?** | Where did the trainees receive the shuttlecock?  Where did the trainees receive the brush? |
| *Mobile*  *Trowel* | **How did the patient contact the doctor?**  **What did the bricklayer use to lay cement?** | **What did the bricklayer use to lay cement?**  **How did the patient contact the doctor?** | **What was the employee given to use at work?**  **What was the employee given to use at work?** |
| *Water*  *Jam* | **What did the waiter spill?**  **What did granny make for her jam tarts?** | **What did granny make for her jam tarts?**  **What did the waiter spill?** | **What did the campers take on their camping trip?**  **What did the campers take on their camping trip?** |
| *Ribbon*  *Lock* | **What did the girl tie her hair with?**  **What did the cyclist secure his bike with?** | **What did the cyclist secure his bike with?**  **What did the girl tie her hair with?** | **What were the students given in their art class?**  **What were the students given in their art class?** |
| *Shirt*  *Poster* | **What did the maid iron?**  **What did the advertisers stick on the wall?** | **What did the advertisers stick on the wall?**  **What did the maid iron?** | **What was the gentleman handed?**  **What was the gentleman handed?** |
| *Letter*  *Medicine* | **What did the woman post?**  **What did the nurse give her patient?** | **What did the nurse give the patient?**  **What did the woman post?** | **What did the customer request?**  **What did the customer request?** |
| *Cell*  *Hotel* | **Where was the criminal locked up?**  **Where did the couple stay?** | **Where did the couple stay?**  **Where was the criminal locked up?** | **Where did the couple visit?**  **Where did the couple visit?** |
| *Thunder*  *Sun* | **What did they see from their bedroom window?**  **What was the weather like in summer?** | **What was the weather like in summer?**  **What did they see from their bedroom window?** | **What did they wake up to?**  **What did they wake up to?** |
| *Envelope*  *Nest* | **What did he put the stamp on?**  **Where did the birds lay their eggs?** | **Where did the birds lay their eggs?**  **What did he put a stamp on?** | **What did they pick up?**  **What did they pick up?** |
| *Umbrella*  *Watch* | **What did he use during the rainstorm?**  **Where did he check the time?** | **Where did he check the time?**  **What did he use during the rainstorm?** | **What did the brother take with him?**  **What did the brother take with him?** |
